# Arthroscopic rod technique compared to stress ultrasound in the dynamic evaluation of lateral ligament instabilities of the elbow

**DOI:** 10.1007/s00402-022-04491-5

**Published:** 2022-07-11

**Authors:** Johannes Plath, Alexander Otto, Stefan Förch, Sebastian Siebenlist, Bianca Grosser, Edgar Mayr, Andreas B. Imhoff, Andreas Lenich

**Affiliations:** 1 Department of Trauma, Orthopaedic, Plastic and Hand Surgery, University Hospital of Augsburg, Stenglinstrasse 2, 86156 Augsburg, Germany; 2 Department of Orthopedic Sports Medicine, Klinikum Rechts Der Isar, Technische Universitaet Muenchen, Muenchen, Germany; 3 Department of Pathology, University Hospital of Augsburg, Augsburg, Germany; 4 Zentrum Für Ellenbogen- Und Schulter-Therapie (ZEST), Orthopädie Am Stiglmaierplatz, Munich, Germany

**Keywords:** Elbow instability, Arthroscopy, Instability testing, Switching rod

## Abstract

**Introduction:**

The purpose was to compare the arthroscopic rod technique to stress ultrasound in the dynamic assessment of lateral elbow instabilities.

**Materials and methods:**

Fifteen elbows of eight specimen with a mean age of 66.4 ± 13.3 years were assessed in a prone position following a defined dissection setup. After evaluation of the native status, an arthroscopic dissection of the radial collateral ligament (RCL) or lateral ulnar collateral ligament (LUCL), and finally of entire capsuloligamentous structures was performed. Three raters examined each state (native, RCL or LUCL lesion, complete lesion) with the arthroscopic rod technique in 90° flexion and with stress ultrasound in 30 and 90° flexion. The intra-class correlation coefficient (ICC) was calculated to assess the interrater reliability as well as test–retest reliability for each testing modality (arthroscopy and ultrasound).

**Results:**

The arthroscopic rod technique showed a superior interrater and test–retest reliability of 0.953 and 0.959 (*P* < 0.001), respectively, when compared to stress ultrasound with an ICC of 0.4 and 0.611 (*P* < 0.001). A joint space opening during arthroscopy of > 6 mm humero-ulnar or > 7 mm humero-radial was indicative for a lateral collateral ligament lesion. However, a differentiation between an isolated RCL or LUCL tear was not possible. A lateral joint opening of ≥ 9 mm was only observed in complete tears of the lateral capsuloligamentous complex.

**Conclusions:**

The arthroscopic rod technique showed a superior interrater and test–retest reliability when compared to stress ultrasound. Arthroscopic assessment for radial elbow instability was found to be reliable and reproducible. A joint gapping ≥ 9 mm in the arthroscopic evaluation is a sign for a complete insufficiency of the radial capsuloligamentous complex. However, it is not possible to precisely distinguish between a lesion of the RCL or LUCL by arthroscopy.

On the basis of our results, dynamic ultrasound testing may be inappropriate to objectify lateral elbow instability.

## Introduction

With an incidence of 5.2 per 100.000, elbow dislocations are the second most common dislocations in the human body. In most cases, young adults, who are active in sports, are affected [1]. Despite the generally favorable long-term results after conservative therapy, chronic subtle joint instabilities can persist after a traumatic dislocation and may lead to pain, subjective instability or even movement restrictions in this young and active patient population [2–6]. Furthermore, apart from acute trauma, chronic elbow instabilities may also be the result of a chronic epicondylitis, repetitive corticosteroid injections and surgical approaches [2, 3, 7–9].

The precise identification of elbow instabilities in the chronic setting represents a great challenge, even for the experienced orthopedic surgeon. Especially in unsedated patients, the clinical evaluation of the elbow is infringed and the interpretation of an instability severity is not reliable [10]. This may result in overlooking ligamentous instability of the elbow [2, 3].

Multiple imaging modalities can be applied for the diagnosis of elbow instability [11]. Magnetic resonance imaging (MRI) has been established as gold standard for diagnosing soft tissue injuries of the elbow [12]. However, MRI remains a static examination of a dynamic problem and may be inappropriate to assess dynamic aspects of instability [13, 14].

Stress ultrasound is used by many clinicians to evaluate dynamic elbow stability and represents a fast as well as non-invasive modality with greater availability and high imaging resolution [12, 14–16].

The value of arthroscopy in the diagnosis as well as treatment of chronic elbow instability has recently increased [17–19].

As early as 1996, Field et al. [20] introduced a technique that used switching rods to evaluate elbow stability on the medial side in cadaveric specimen. Different rod sizes were used to objectively quantify the gapping of the medial compartment under valgus-stress. A complete release of the medial collateral ligament led to a significant medial joint opening of 4 to 10 mm. Later, the “elbow drive-through sign” was described by several authors during elbow arthroscopy [21–23]. Here, the complete insufficiency of the lateral collateral ligaments allows the surgeon to arthroscopically glide from the lateral compartment to the medial compartment.

In our experience, arthroscopic stability testing using a scaled switching rod is a valuable tool to assess elbow instabilities. To our knowledge, no standardized procedure has been described to arthroscopically objectify a lateral capsuloligamentous complex insufficiency. Furthermore, the arthroscopic assessment has not yet been compared to stress ultrasound as the second dynamic instability assessment modality.

The purpose of this study was to compare arthroscopic evaluation with scaled switching rods to stress ultrasound. It was hypothesized that the arthroscopic evaluation provides a more precise assessment with a higher reliability when compared to stress ultrasound.

## Materials and methods

Eight male Specimen with a mean age of 66.4 ± 13.3 (range 48–83) years were assessed for elbow instability. Each specimen included paired elbows of a complete human torso provided by the pathologic institute of the University Hospital of Augsburg. Prior to testing, it was ensured that the specimen had no history of elbow surgery and an assessment with a mobile C-arm image intensifier was performed to exclude severe osteoarthritis. One elbow was excluded due to osteoarthritis, which resulted in seven right and eight left elbows that were finally assessed. This study was reviewed and approved by the ethical board of the University Hospital of Augsburg (IRB No. 2017-26).

### Testing setup and arthroscopic dissection

**Fig. 1:**
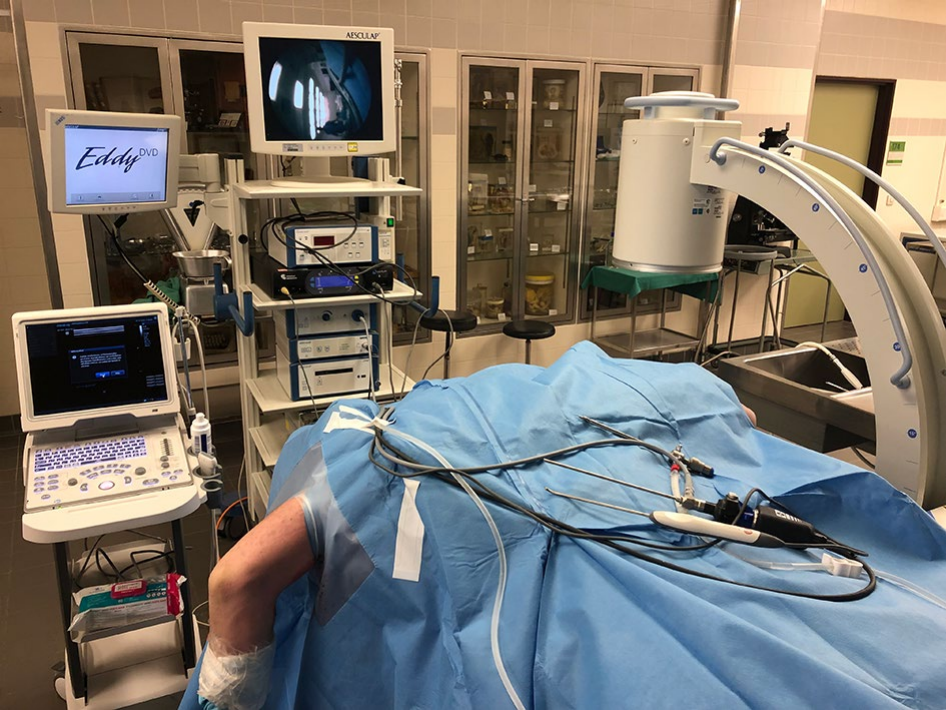
Study setup and positioning of the cadaver in prone position. The humerus was secured to a mount

Testing was performed with the specimen thawed to room temperature and free range of motion of the elbow joint. It was ensured that the range of motion was not infringed due to cadaveric rigidity. The cadaver was placed in prone position and the humerus was secured to a mount allowing the desired flexion angle of the elbow joint (Fig. 1).

Standardized portals orientated on anatomic landmarks were established in every specimen. A low posterolateral portal at the soft-spot between the radial epicondyle, the radial head and the tip of the olecranon was used for initial joint distension. Before instability testing, it was ensured that a thorough arthroscopy was possible and a complete resection of the posterolateral plica was performed.

**Fig. 2:**
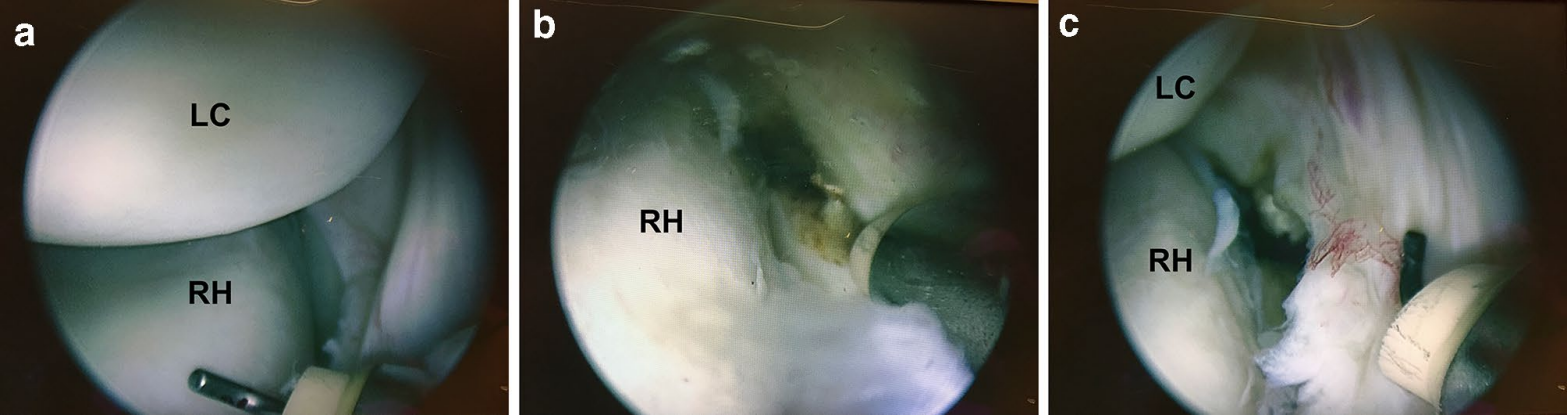
Arthroscopic view via a high posterolateral portal visualizing the posterolateral elbow compartment of a left elbow (*RH* radial head, *LC* lateral condyle). View on the intact lateral capsuloligamentous complex of the elbow (**a**), dissection of the capsule and ligaments with a hook electrode (**b**) and incised anterior capsuloligamentous structures of the elbow (**c**)

After evaluation of the native status, selective arthroscopic dissection of the radial collateral ligament (RCL) or the lateral ulnar collateral ligament (LUCL), respectively, was performed. The order of RCL or LUCL dissection was determined by a simple randomization technique. Finally, the complete lateral ligamentous complex was cut and complete lateral instability was reached. The arthroscopic dissection was adapted from McAdams et al. [8] (Fig. 2a–c).

Each state: native (*n* = 15), RCL lesion (*n* = 7) or LUCL lesion (*n* = 8), and complete lateral ligamentous lesion (*n* = 15) was assessed with arthroscopy and ultrasound by three independent examiners to enable interrater reliability calculations. All examiners were orthopedic surgeons specialized in arthroscopic surgery. For test–retest reliability calculations, all measurements of the senior author (A.L.) were repeated at a later stage during testing.

### Arthroscopic testing

Visualizing through a high posterolateral portal lateral to the tip of the olecranon stability testing was performed. The low posterolateral portal was used to enter the joint with rods of different sizes. The rods were gently inserted to avoid any damage to the cartilage beginning with a diameter of 1 mm and increasing in increments of 1 mm up to a maximum of 9 mm.

**Fig. 3:**
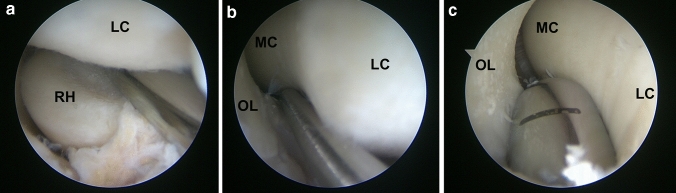
Instability testing of a left native elbow for humero-radial stability at the center of the radial head (**a**) and for humero-ulnar stability at the level of the incisura olecrani (**b**) fitting a 4 mm switching rod. After dissection of the lateral collateral ligament a 9 mm rod fits at the level of the incisura olecrani (**c**). (*RH* radial head, *LC* lateral condyle, *MC* medial condyle, *OL* olecranon)

Humero-radial instability was assessed at the center of the radial head and humero-ulnar instability at the level of the incisura olecrani. The largest diameter that could be fitted into the joint space without excessive stress was recorded. (Fig. 3a–c) All stability measurements were performed with the elbow flexed at 90 degrees and at neutral forearm rotation.

### Ultrasound

**Fig. 4:**
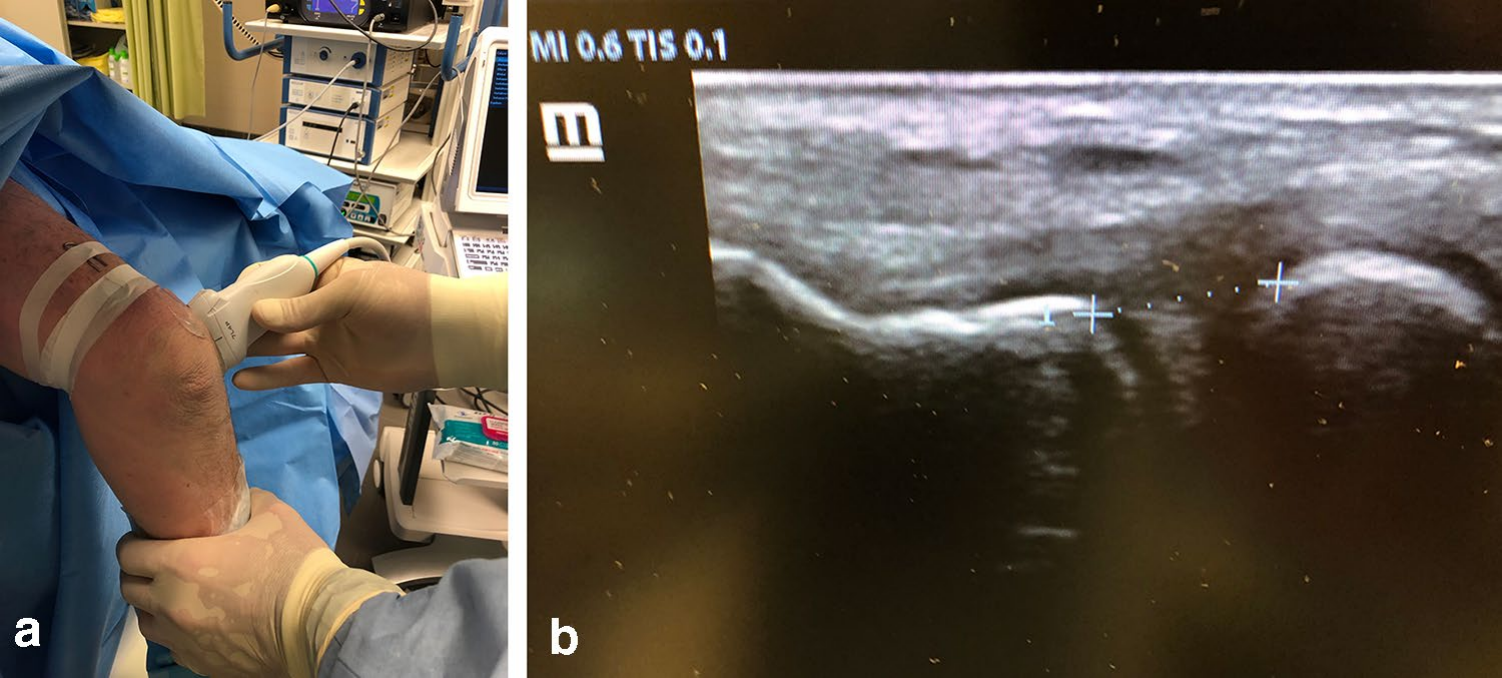
Ultrasound assessment of lateral stability. The probe was aligned to the lateral epicondyle and the extensor tendons (**a**). The shortest distance between the rim of the radial head and the capitellum was measured (**b**)

In the ultrasonic evaluation (Mindray Z6, Mindray, Shenzhen, China), the transducer was aligned to the lateral epicondyle and the extensor tendons. The shortest distance between the rim of the radial head and the capitellum was documented. (Fig. 4a, b) All measurements were performed at 30 and 90° elbow flexion with neutral forearm rotation. The difference between the varus loaded vs. unloaded elbow was documented. It was ensured that the ultrasound probe maintained its orientation as well as position during the varus maneuver to avoid deviations in the plane of measurement.

### Statistics

Statistical analysis was performed using SPSS software version 23 (SPSS Inc). Based on the observations of Field et al. [24], an a priori power analysis was performed. It was determined that a sample size of 15 would provide a power of 80% to detect a 2 mm difference between the native and the sectioned state at an alpha of 0.05.

The intra-class correlation coefficient (ICC) overall as well as for each dissection state was calculated to assess the interrater reliability as well as test–retest reliability for each testing modality (arthroscopy and ultrasound).

Absolute values for joint space opening were calculated for each dissection state and presented as means ± SDs and ranges.

## Results

The successive dissection of the lateral ligamentous structures resulted in an increase in lateral instability in both testing modalities. Overall, arthroscopy showed a superior interrater and test–retest reliability of ICC 0.953 and 0.959 when compared to sonography with an ICC of 0.4 and 0.611, respectively. This observation was independent from the degree of lateral instability. (Table 1).

**Table 1:** Interrater-correlation-coefficient (ICC) for interrater and test–retest reliability at lateral ligament dissectioning

lateral ligament dissection	Arthroscopy			Ultrasound		
	IR-ICC	TrT-ICC	*p*-value	IR-ICC	TrT-ICC	*p*-value
Intact	0.959	0.955	0.0001	0.068	0.378	0.0001
LUCL tear	0.942	0.945	0.0001	− 0.043	0.531	0.0001
RCL tear	0.945	0.946	0.0001	− 0.087	0.166	0.0001
Complete lateral tear	0.951	0.951	0.0001	0.427	0.562	0.0001

*ICC* interrater-correlation-coefficient, *IR* interrater, TrT test–retest, *RCL* radial collatera ligament, *LUCL* lateral ulnar collateral ligament

Despite the high interrater and test–retest reliability for the arthroscopic rod technique, we found a high inter-individual variance for lateral joint opening at an intact lateral ligament complex with a mean of 4 mm (range 2–6) for humero-ulnar and 3.7 mm (range 2–7) for humero-radial testing. A high inter-individual variance was also observed after sequential dissection of the lateral collateral ligament complex. (Table 2) However, a joint space opening of more than 6 mm humero-ulnar or more than 7 mm humero-radial was indicative for a lateral collateral ligament lesion. On the basis of our data, a differentiation between an isolated RCL or LUCL tear by absolute values was not possible.

**Table 2:** Absolute values of lateral joint opening during arthroscopic and ultrasound measurements at lateral ligament dissectioning

lateral ligament dissection	Arthroscopy			Ultrasound		
	Position	Mean ± SD	Range	Position	Mean ± SD	Range
Intact	Humero-ulnar	4 ± 1,228	2–6	30°	0,69 ± 1,039	− 0,7–2,6
	Humero-radial	3,7 ± 1,266	2–7	90°	0,58 ± 1,12	− 1,7–4,3
LUCL tear	Humero-ulnar	6 ± 1,291	4–8	30°	0,73 ± 1,047	− 0,9–3,6
	Humero-radial	5,2 ± 1,707	4–8	90°	1,18 ± 1,41	− 3,4–3,4
RCL tear	Humero-ulnar	6 ± 1,149	4–7	30°	1,82 ± 2,013	− 1,8–6,6
	Humero-radial	6,1 ± 1,161	4–8	90°	1,63 ± 1,854	− 0,9–7.9
Complete lateral tear	Humero-ulnar	8,7 ± 0,564	7–9	30°	3,33 ± 2,67	− 0,8–12,7
	Humero-radial	8,7 ± 0,491	7–9	90°	2,42 ± 1,745	− 0,6–9,2

*RCL* radial collatera ligament, *LUCL* lateral ulnar collateral ligament

A lateral joint opening of 9 mm or more at the humero-ulnar or humero-radial compartment, however, was only observed at complete tear of the lateral capsuloligamentous structures (Table 2).

## Discussion

The most important finding was that a high interrater reliability as well as test–retest reliability was observed for the arthroscopic stability assessment. Even in the setting of multiple insufficient ligaments, the arthroscopic assessment provided a high interrater and test–retest reliability in the current study. The ultrasound assessment, however, showed poor measurement reliabilities.

To correctly treat our patients, a precise identification of the affected ligaments in chronic elbow instability is mandatory, yet challenging [24]. A reproducible as well as simple test modality closest to clinical application would allow optimizing surgical treatment and avoid missing relevant ligamentous instabilities. Here, the arthroscopic assessment of the elbow with rods has been introduced [21–24]. However, the accuracy of an arthroscopic evaluation is unknown [25].

We could observe a reliable and reproducible assessment of lateral elbow instability through the arthroscopic rod technique. Despite a high interrater as well as test–retest reliability for this technique, we found a high inter-individual variance for lateral joint opening in the intact ligamentous state. As observed by McAdams et al. [8], we were also unable to specifically differentiate between isolated RCL and LUCL tears. However, if the surgeon finds a joint gapping of more than 6 mm humero-ulnar, or more than 7 mm humero-radial during elbow arthroscopy, respectively, the surgeon must assume a lesion of the lateral collateral ligament complex. Moreover, if the surgeon observes a joint gapping of more than 9 mm all lateral capsuloligamentous structures are torn. In this scenario, a lateral collateral ligament reconstruction is indicated.

For ultrasound assessment of elbow instability, the interpretation and application were previously reported to be highly dependent on the experience of the examiner [25]. Furthermore, to our knowledge, no standard for evaluating the lateral ligamentous complex with ultrasound exists [26]. However, dynamic ultrasonography can provide a unique view of the radial head subluxation [27]. In the current ultrasound assessment, the elbows were examined at two different flexion angles (30 and 90° elbow flexion) to evaluate the anterior (RCL) or posterior (LUCL) parts of the lateral collateral ligament complex. Here, stress ultrasound resulted in a high variability with low ICC. Despite the usefulness of applying stress ultrasound for the differentiation between a stable and unstable elbow joint, the inconsistent findings make it impossible to objectifying the severity of instability. The inconsistency of data becomes even more obvious when appreciating the high data scattering for ultrasound assessment. A key issue could be—despite efforts to avoid this bias—the loss of the initial measurement plane of the ultrasound transducer due to elbow joint instability, while stress was applied. The effort to find the shortest distance on a correct measuring plane again may explain the tendency to lower mean joint gapping for ultrasound. This underlines the importance to develop a standardized ultrasound stress evaluation protocol which is orientated on clear anatomic landmarks and is robust to stress application. Furthermore, in clinical practice, muscular contraction during testing may further bias instability assessment.

There are limitations to our study that should be considered when interpreting the results. As delineated in the section above, the ultrasonic assessment might have been influenced by deviations in the measurement plane. This might be a disadvantage of our clinical testing setup. However, the variability of the ultrasonic evaluation represents an issue that can occur in the daily clinical examination. The authors preferred to apply a setup closest to their actual clinical setting to achieve a higher clinical relevance. The importance of maintaining transducer stability during sonographic testing has been stressed previously by Camp et al.[15, 28] during sonographic posterolateral rotatory instability testing. The authors suggested video capturing and distance measurements of joint opening on the recorded footage. This would minimize the loss of the measurement plane during instability testing. Furthermore, joint instability was currently evaluated at the humero-ulnar and humero-radial part of the elbow joint to differentiate the exact location of the lesion. However, despite a standardized approach, the anatomy of the lateral collateral ligament of the elbow may be too complex to be evaluated by these two simple measurements. Moreover, arthroscopic testing was only performed at 90° of flexion as sufficient arthroscopic visualization of the posterolateral joint closer to extension was not possible. As mentioned above, the aim of the study was to compare common dynamic testing setups as routinely performed in clinical practice.

A strength of this study is that a test setup closest to the actual clinical setting was applied, which allowed to increase the clinical relevance of this study. Another strength of the current setup is that two measurements were performed in each modality to assess the elbow humero-ulnar as well as humero-radial. The anatomic dissection provided a plausible effect on the elbow joint stability, which was measurable with both modalities.

## Conclusion

The arthroscopic rod technique showed a superior interrater and test–retest reliability when compared to stress ultrasound. Arthroscopic assessment for radial elbow instability was found to be reliable and reproducible. A joint gapping ≥ 9 mm in the arthroscopic evaluation is a sign for a complete insufficiency of the radial capsuloligamentous complex. However, it is not possible to precisely distinguish between a lesion of the RCL or LUCL by arthroscopy.

On the basis of our results, dynamic ultrasound testing may be inappropriate to objectify lateral elbow instability.
